# Radiation-Induced Brain Injury: Age Dependency of Neurocognitive Dysfunction Following Radiotherapy

**DOI:** 10.3390/cancers15112999

**Published:** 2023-05-31

**Authors:** Claudia E. Rübe, Silvia Raid, Jan Palm, Christian Rübe

**Affiliations:** Department of Radiation Oncology, Saarland University Medical Center, Kirrbergerstrasse Building 6.5, 66421 Homburg, Germany

**Keywords:** ionizing radiation, radiotherapy, radiation-induced brain injury, neurocognitive dysfunction, age dependency

## Abstract

**Simple Summary:**

Radiation therapy-related brain damage with neurocognitive impairment is a common long-term side effect in cancer survivors and significantly impairs the quality of life. Increasing evidence indicates the increased vulnerability of the developing brain to the neurotoxic effects of ionizing radiation (IR). In this review, historical and current clinical evidence on the age dependency of radiation-induced neurocognitive dysfunction is summarized. Moreover, recent research developments regarding the mechanistic causes for this age-related extent of brain damage following IR exposure are presented.

**Abstract:**

Cranial radiotherapy is a known risk factor for neurocognitive impairment in cancer survivors. Although radiation-induced cognitive dysfunction is observed in patients of all ages, children seem to be more vulnerable than adults to suffering age-related deficits in neurocognitive skills. So far, the underlying mechanisms by which IR negatively influences brain functions as well as the reasons for the profound age dependency are still insufficiently known. We performed a comprehensive Pubmed-based literature search to identify original research articles that reported on age dependency of neurocognitive dysfunction following cranial IR exposure. Numerous clinical trials in childhood cancer survivors indicate that the severity of radiation-induced cognitive dysfunction is clearly dependent on age at IR exposure. These clinical findings were related to the current state of experimental research providing important insights into the age dependency of radiation-induced brain injury and the development of neurocognitive impairment. Research in pre-clinical rodent models demonstrates age-dependent effects of IR exposure on hippocampal neurogenesis, radiation-induced neurovascular damage and neuroinflammation.

## 1. Introduction

Ionizing radiation (IR) has become an indispensable tool of modern health care. The growing use of IR as a diagnostic and therapeutic tool in clinical medicine raises the question of possible health effects. The generally relatively low doses used in current diagnostic imaging modalities have been shown to increase the risk for brain cancers in children [1] but have no noticeable effect on neurocognitive functions. In radiation oncology, by contrast, the standard of care for primary and metastatic brain tumors includes high-dose radiation to the skull, and 50–90% of long-term survivors develop disabling cognitive dysfunction later on. The exact pathomechanisms of the radiation-cognitive syndrome are still insufficiently researched and to date, there is neither an effective prevention nor an efficient long-term treatment. Here, the current knowledge about the impact of IR on neurocognitive functions is described, especially in relation to age dependency.

## 2. Methods

In alignment with best practices in search methodology, the PubMed database was used to retrieve comprehensive sets of relevant English-language articles using combinations of search terms (ionizing radiation/irradiation/radiotherapy/cognitive function/neurocognitive impairment/scalp irradiation/mental retardation/intellectual quotient (IQ)/intellectual deficit). From these publications (180 studies in humans in total), all studies examining the age dependency of radiation-induced neurocognitive dysfunction were selected. Only studies that provided sufficient information to assess an age-related impairment of neurocognitive function were considered (37 studies). The following information was listed for the selected studies: authors ‘names, year of publication, study population (including the country), number of participants, type of IR exposure, age at IR exposure, brain dose as part of IR exposure, outcome following IR exposure, age at outcome measurement.

## 3. Results of Clinical Studies on Humans

### 3.1. Radiation Effects of Low-to-Moderate Doses on Neurocognitive Development

Evidence of the effects of IR on the developing human brain was first documented in children of atomic-bomb survivors in Japan, who were exposed prenatally (during the first and second trimesters of pregnancy) to low-to-moderate doses and revealed mental retardation [2,3,4,5,6,7,8,9] (Table 1). However, atomic-bomb survivors exposed during their adolescence (aged ≥13 years at the time of bombings) did not show any deleterious effect on late-life cognitive function in adulthood [10]. Moreover, no increased risk of premature neurodegeneration was observed among aging atomic-bomb survivors exposed in-utero or during early childhood [11,12,13,14]. These studies of atomic-bomb survivors suggest that the long-term effects of low-to-moderate radiation exposure on late-life neurocognitive function are limited (Table 1). Potential cognitive consequences of low-dose radiation exposure from environmental disasters such as the Chernobyl accident have been intensely debated over the last decades [15]. Despite numerous publications on potential health effects during gestation, childhood and adolescence, there is no clear evidence that the low-dose fallout from Chernobyl increased the risk for neurocognitive dysfunction [16,17].

Consequences of post-natal radiation exposure were also studied in children treated by X-ray epilation for tinea capitis. Studies of the American and Israeli tinea capitis cohorts evaluating thousands of children up to 20 years after IR exposure (mean doses 1.3–1.5 Gy) could demonstrate lower IQ scores with poorer school performance and higher frequencies of mental diseases compared to non-irradiated children [18,19] (Table 2). During 1950–1960 Swedish boys received IR for cutaneous hemangiomas before the age of 18 months and their cognitive abilities were analyzed by military test scores at the age of 18 years. This large Swedish cohort study indicates that even low-level exposure of the infant brain may adversely affect intellectual development [20]. Repeated analysis of this Swedish cohort suggests that particularly the hippocampal dose is a good predictor of late cognitive side effects [21] (Table 2). On the other hand, the very low doses generally used in diagnostic procedures do not seem to have any noticeable effect on neurocognitive function. Accordingly, IR exposure from pelvimetric examination in-utero had no detectable effects on children’s final primary school grades [22]. Moreover, head CT examination at the age of 6–16 years does not seem to affect later cognitive functions [23].

### 3.2. Radiation Effects on Neurocognitive Function in Brain Cancer Survivors

Further evidence for radiation-induced cognitive impairment has come from studies on survivors of childhood, adolescent, or adult cancer. Radiotherapy (RT) is an indispensable treatment mainstay for most primary brain tumors and for brain metastases originating from extracranial tumors [24]. Brain RT is subdivided into whole-brain radiotherapy, in which the entire brain and brainstem are irradiated, and partial-brain radiotherapy, which includes treatment of the tumor or tumor bed and surrounding margin. In modern radiation oncology, different techniques of conformal radiotherapy are employed to deliver high doses to the tumor of cancer patients, while limiting the dose to surrounding healthy tissues to avoid adverse toxicities. With intensity-modulated radiotherapy (IMRT, stop-and-shoot or rotational arc techniques) multiple photon beams from different directions and with adjusted intensities permit close shaping of radiation dose to target volumes, thereby delivering high doses to tumors while sparing healthy brain tissue. Stereotactic radiosurgery relies on precise three-dimensional (3D) imaging and localization to deliver ablative doses of radiation to small tumors (≤3 cm in diameter) with minimal impact on the surrounding healthy brain. In addition to these highly conformal techniques based on external photon beams, proton therapy is increasingly used, especially for treating pediatric brain tumors [25].

RT is an effective treatment method for patients of all ages with different types of brain tumors. According to the timing of clinical symptoms, radiation-induced brain damage can be characterized as acute, early delayed, and late injury (even if these early side effects only rarely occur with modern radiation techniques). Acute microvascular damage with cerebral edema can develop in hours to days after high doses to the brain. Early delayed brain damage occurs within the first few months after IR exposure and can result in transient demyelination lesions, followed by dysfunction of neural networks in these affected brain regions [26]. However, these early, sometimes quite impressive manifestations of brain damage are considered temporary and reversible. In contrast, the late manifestations of brain damage in both white and gray matter areas are often persistent and progressive and can ultimately lead to brain necrosis. These severe parenchymal defects are accompanied and exacerbated by vascular damage leading to impaired perfusion and usually begin to occur 4–6 months post-IR [27]. Late brain injury can develop progressively even years after IR exposure and the organic damage with correspondingly different neurocognitive deficits is generally irreversible [28].

Radiation-induced cognitive dysfunction is a complex of symptoms characterized by a reduction in the intelligence quotient (IQ) and impairments of core functions, such as attention, vigilance, working memory, executive functions, psychomotor performance, visual-motor integration, speed of information processing, or learning deficits. These core deficits can be associated with behavioral changes and can compromise social and academic performance and quality of life. In the past, potential neurocognitive morbidity after IR exposure was difficult to measure because neurocognitive testing was often limited by the lack of standardized and validated examination methods. In addition, given the overall reduced patient compliance, neurocognitive status was often not recorded before the start of the radiation treatment. Only in more recent studies, comprehensive neurocognitive and quality-of-life assessments were conducted at baseline and at follow-up [29].

#### 3.2.1. Childhood Cancer Survivors

Childhood cancer survivors often suffer from cognitive dysfunction, which can occur years after radiotherapy for pediatric brain tumors or acute lymphocytic leukemia (ALL) [30,31]. In childhood brain tumors, most reports of radiotoxicity have come from survivors of low-grade gliomas or medulloblastoma, the most frequently observed brain tumors in children with a good prognosis [32,33]. Children who receive radiation therapy for their brain tumors have a greater risk for cognitive impairment than those who undergo surgery and/or chemotherapy alone [34]. The level of the total dose and the extent of the radiation field is strongly associated with the later development of cognitive dysfunction [35,36]. The reduction in the total dose and volume of cranial radiation while intensifying chemotherapy has improved survival and reduced the extent of neurocognitive impairment [37,38]. Because of these treatment modifications, the prevalence and severity of neurocognitive dysfunction in childhood cancer survivors have declined over the past several decades [30]. Young age at radiation treatment is the most important patient-related risk factor for neurocognitive impairment, due to radiation-induced brain damage in a particularly vulnerable phase of neuronal development [39,40,41,42,43,44,45] (Table 3). However, in the case of proton RT or modern conformal photon RT, this clear age-dependent effect of radiation-induced brain damage is no longer evident [45,46,47,48] (Table 3).

After the introduction of prophylactic whole-brain RT in pediatric patients with leukemia, it became apparent that radiation leads to IQ reductions, particularly in younger children [36,49,50,51,52,53,54,55] (Table 4). As evidence emerged that whole-brain RT in ALL was associated with IQ decrease, the total dose of cranial radiation was gradually reduced since the 1980s. Nowadays, whole-brain RT is generally avoided in children with leukemia [56,57]. An actual meta-analysis of children and adolescent survivors of ALL demonstrated clinically significant differences in cognitive functions, with lower scores of total IQ, verbal and performance IQ compared to healthy controls [58]. Moreover, recent studies suggest that adult survivors of childhood cancer treated with prophylactic whole-brain RT have a higher risk of developing dementia later in life [59]. Aging survivors of ALL who received 24 Gy (but not 18 Gy) whole-brain RT revealed early-onset memory loss with reduced ability to recall verbal associations and reproduce visual patterns [59]. Functional neuroimaging of these survivors with cognitive impairment demonstrated reduced structural integrity of anatomical regions established for memory formation [59]. Longitudinal studies of adult survivors of childhood medulloblastoma suggest that RT causes not only neurocognitive late effects throughout the lifespan of children and adolescents but may even progress for decades after treatment has been completed [41]. According to this study, RT is associated with the progressive decline in working memory already at different ages throughout adulthood lifespan, reflecting a common sign of cognitive aging [41]. Collectively, these findings suggest that survivors of childhood cancer who received cranial RT with higher doses may experience early onset of cognitive aging.

#### 3.2.2. Adulthood Cancer Survivors

Brain tumor survivors who received RT as adults may also experience progressive deterioration in neurocognitive functions [60]. High-grade gliomas account for 50% of all primary brain tumors in adults, but because of the often early tumor progression, patients usually do not experience neurocognitive impairment from RT. Most studies evaluating the relationship between RT and cognitive impairment are based on patients with low-grade gliomas. Findings from the literature propose that radiation treatment factors such as total and partial dose, target volume size, and radiation technique define the potential risk of RT-related neurotoxicity [61,62,63,64,65]. However, clinical studies indicate that neurocognitive deficits in patients with brain tumors usually have a complex multifactorial genesis [66,67,68,69]. The causes of cognitive impairment in patients with brain tumors can be very diverse and include tumor-related factors (location, size and growth behavior of the tumor), treatment-related factors (neurosurgical interventions, use of antiepileptic drugs, parenteral or intrathecal chemotherapy) and patient-related factors (age during treatment, pre-existing comorbidities). Brain tumor patients often have to contend with significant neurological symptoms that severely impair not only their cognitive functions but also their quality of life. Neurosurgical resection of brain tumors is often required to provide histopathological specimens and to reduce tumor burden. The size and exact location of the tumor in the brain determines the extent of the required resection and thus largely determines the risk of complications. While a complete resection increases the chance of long-term survival, the benefits of an aggressive resection must be weighed against the risk of potentially severely disabling brain damage.

Over the past twenty years, the use of 3D conformal RT has led to a reduction in the amount of brain tissue exposed to high doses. In this regard, the results of most prospective studies suggest limited harm from focal RT and support the hypothesis that cognitive impairment in adult patients is mainly due to tumor recurrence.

Whole-brain RT in adult tumor patients is used to prevent or delay the spread of cancer cells to the brain. Prophylactic cranial radiotherapy was the standard care for patients with small-cell lung cancer, showing a complete response to front-line chemotherapy. However, recent clinical trials suggest that prophylactic whole-brain RT did not provide survival benefits, but an increased risk of neurocognitive decline that can affect quality of life [70]. Allogeneic bone marrow transplantation for hematological malignancies generally requires total-body irradiation to eradicate malignant cells (in sanctuary organs that are not reached by chemotherapeutic drugs) and to induce immunosuppression to prevent the rejection of donor marrow. Clinical studies with neuropsychological testing of adult patients indicate that total-body irradiation with doses ≥12 Gy can lead to cognitive deficits in long-term survivors [71].

### 3.3. Summary of Clinical Trials in Humans

Overall, there is a clear age dependency on radiation-induced brain damage. In the vulnerable phases of pre- and post-natal brain development, even single exposures with moderate doses of IR (0.1–2 Gy) have negative long-term effects on neurocognitive functions. While moderate doses have serious consequences for children’s brain development, the influence of very low doses (≤0.1 Gy) on the neurocognitive outcome is less clear and no negative effects could be proven so far. The interpretation of data concerning neurocognitive deficits in cancer survivors is often difficult, due to the nearly impossible differentiation between adverse side effects of radiotherapy from those caused by underlying cancer disease, concomitant tumor therapy, as well as other influencing factors. Therefore, data obtained from survivors of childhood cancer treated with prophylactic whole-brain RT are by far the most conclusive. Numerous studies have shown that the severity of radiation-induced cognitive dysfunction in children and adolescent survivors of acute leukemia is clearly dependent on age (Table 4>). Moreover, recent findings suggest that adult survivors of ALL who received cranial radiotherapy during early childhood have an increased risk of accelerated cognitive aging with developing dementia earlier in life [59]. But even the mature brain of adult tumor survivors can be damaged depending on the irradiated region and various irradiation parameters. So far, there is no evidence for age-related differences in radiation-induced neurocognition effects in the mature adult brain. Moreover, there is no knowledge about whether brain IR in adulthood may lead to premature neurodegeneration in old age. Some preclinical studies suggest that radiation-induced senescence may lead to premature brain aging and may predispose to neurodegenerative disorders including Alzheimer´s and Parkinson´s disease [72,73].

## 4. Results of Pre-Clinical Studies in Animal Models

### 4.1. Elucidating the Mechanisms of Radiation-Induced Brain Injury Using Rodent Models

The genesis of radiation-induced brain injury with the development of neurocognitive decline is highly complex with multiple molecular and cellular mechanisms interacting at different levels in various brain compartments. Historically, clinical studies focused on radiation-induced brain tissue changes appearing over months to years after radiotherapy (such as white matter deterioration detectable by diagnostic imaging) since no more sensitive analytical methods were available. However, increasingly many studies revealed that CNS alterations and cognitive dysfunction develop much earlier than 6 months following IR exposure [74]. Technical improvements in imaging modalities along with enhanced experimental techniques in animal- and cell-based model systems have been able to reveal subtle evidence of damage to different neuroanatomical domains already in the first days and weeks after IR exposure [74]. This has led to the current hypothesis that relatively subtle early forms of radiation-induced brain damage can trigger a chronic pathophysiology leading to permanent cognitive decline and possibly escalating to dementia [74]. Accumulating evidence from animal models suggests that cognitive decline following IR exposure involves radiation-induced damage in multiple cell populations, causing structural and functional alterations simultaneously in different neuronal lineages, in supporting glial cells, as well as in cerebral microvasculature. Immediately after IR exposure, injury-related interacting processes are set in motion that alter the signaling environment in the stem cell niche of the hippocampus, a brain structure critical to short-term memory and learning. Neurophysiological disturbances may progressively alter neuronal stem cell niches and changed niche conditions may lead to reduced neurogenesis with pathophysiological effects on cognitive function [75]. Overall, this multifactorial scenario with neurovascular and neuroinflammatory responses may result in the depletion and long-term dysfunction of neurons, and consequently in permanent cognitive impairment.

In the central nervous system (CNS), multiple subtypes of neurons are interconnected to maintain the functionality of the complex mammalian brain. Glial cells, categorized into lineages of microglia, astrocytes, and oligodendrocytes, collectively support neuronal viability and functionality. In the last decades, the diverse functions and special importance of the different glial cell populations for brain homeostasis under physiological and pathological conditions have been worked out. Astrocytes are the most common cell population in the brain and perform numerous neuroprotective tasks, from energy supply, axon guidance, and synaptic transmission of impulses, to the control of the blood-brain barrier [76]. Microglia cells are specialized immune cells of the brain with phagocytic and antigen-presenting capabilities for the rapid removal of pathogens, apoptotic cells, and cellular debris [77]. Activated microglia undergo morphological changes with modified protein secretion, releasing pro- or anti-inflammatory mediators depending on the situation. The main function of oligodendrocytes is the formation of myelin sheaths around neuronal axons, which ensures rapid signal transmission. In the brain, most proliferating cells outside of stem cell niches are the progenitor cells of oligodendrocytes. Even after low doses of radiation, the oligodendrocyte precursors go into apoptosis, which then leads to progressive demyelination a few weeks to months later [78]. Overall, in recent years there has been increasing recognition that the diverse and dynamic functions of glial cells control essentially all aspects of nervous system formation and function, from the birth and migration of neurons to the formation of dendrites and axons, and up to the assembly of neuronal circuits. As neuronal circuits mature, certain glial cells fulfill key roles in synaptic communication and plasticity, thereby controlling physiological and pathological brain functions. In particular, not only the cell phenotype but also the stage of differentiation can predispose the fate of the affected cells. Indeed, proliferating cells are generally more radiosensitive and undergo apoptosis at lower dose levels compared to terminally differentiated cells.

Evidently, the pathogenesis of radiation-induced brain injury has a multifactorial genesis and depends on both the latency of cell reactions and the dynamics of structural and functional changes. This complex interplay determines the course of the disease over time and ultimately the severity of the organic brain damage. In the context of the above considerations, there are three main pathophysiological concepts to explain the complex mechanisms underlying age-dependent radiation-induced brain disease. One of these is based on the pathogenic mechanism of hippocampal neurogenesis, and the others are focused on the neurovascular and neuroinflammatory etiology of the disease.

### 4.2. Age-Dependent Effects of IR Exposure on Hippocampal Neurogenesis

The hippocampus is a crucial brain structure for the processing of new information and for spatial orientation. [79]. The hippocampus is divided into the dentate gyrus (DG) and the various sub-regions of the cornu ammonis. The subgranular zone (SGZ), a narrow cell layer located between the granular cell layer and hilus of the DG, contains the neural stem cells, which continuously self-renews by asymmetric division and differentiate into neurons and glia cells in a process called *adult neurogenesis* [80]. During their post-mitotic maturation, these neuroprogenitors migrate into adjacent granular cell layers, where they develop their mature morphological and functional properties through the outgrowth of axons and dendrites and thus integrate into established neuronal networks [81]. A growing body of evidence suggests that adult neurogenesis is tightly controlled by environmental conditions in the neurogenic niche composed of various glial cell populations.

Increasing insights from rodent models indicate that IR exposure (even in the moderate and low dose range) impairs hippocampal neurogenesis by eliminating radiosensitive neuroprogenitors and suppressing the differentiation of neuroprogenitors into mature neurons [82]. The age-dependent sensitivity of the developing brain is correlated with the number and vulnerability of neuroprogenitors in the hippocampal stem cell niche [83]. Proliferating neuroprogenitors are inherently more radiosensitive than post-mitotic neurons and IR exposure reduces or ablates hippocampal neurogenesis as the result of massive death of proliferating neuroprogenitor cells. Reduced hippocampal neurogenesis following prenatal irradiation in moderate and low dose ranges is associated with lower cognitive performance as evaluated by behavioral testing [84,85].

According to radiobiological principles dividing cells are more likely to go into apoptosis after radiation damage, the developmental stage of the brain at the time of IR exposure plays an important role in the extent of radiation-induced brain damage [83]. In rodent models, specific radiation-induced effects were observed at different neurodevelopmental stages, suggesting that the biological outcome may differ depending on the timing of IR exposure. Proliferating neuroprogenitors in the embryonic brain are extremely radiosensitive and already react to prenatal IR with 10 mGy in terms of radiation-induced apoptosis [86]. Overall, radiation-induced apoptosis is one of the main mechanisms of neurodevelopmental dysfunction in the context of prenatal in-utero irradiation [84,87,88]. To investigate the influence of low doses of radiation on brain development, mice were exposed prenatally (E11) to IR doses ranging between 0.1 and 1.0 Gy and brain structures and functions were characterized by magnetic resonance (MR) imaging and behavioral testing at 12 weeks of age [89]. Microcephaly with reduced total and regional brain volumes was apparent at doses ≥0.3 Gy. Altered brain functions could be verified by behavioral testing at doses ≥0.5 Gy [84]. Neural progenitors are characterized by specific DNA damage responses, and the damaging effects of IR exposure increase with the proportion of actively proliferating neuroprogenitors that are more susceptible to apoptosis, cell cycle arrest, or premature differentiation [90,91]. Since this relative proportion of proliferating neuropogenitors varies both with regard to developmental stage and specific brain region, this fact explains the increased age-dependent radiosensitivity of circumscribed brain compartments. Repeated exposure to IR can change the fate of neural progenitor cells through increased differentiation into glial cells as part of neurogenesis, with the stem cell pool being reduced over time [92]. For perinatal IR exposure defects in adult neurogenesis were detectable even several months after brain IR and associated with long-term consequences on learning and memory [93,94,95]. Even after postnatal IR exposure with low doses of only 0.1 Gy, long-term changes in the form of reduced neurogenesis with increased apoptosis, disturbed mitochondrial homeostasis, and reduced synaptic plasticity can be observed in the context of hippocampal neurodevelopment [85]. Daily low-dose irradiation (5×, 10×, 15×, 20× fractions of 0.1 Gy) of juvenile and adult mice revealed an accumulation of radiation-induced DNA damage, leading to the progressive decline of hippocampal neurogenesis with reduced numbers of stem/progenitor cell populations and less arborization of dendritic trees that is more pronounced in the immature brain of young animals [96]. In addition, these investigations showed a pronounced shift in the differentiation process of stem/progenitor cells from neurogenesis to gliogenesis [97]. Further evidence supporting the role of neuroprogenitor loss in cognitive dysfunction following IR exposure comes from studies showing that cognitive functions can partially be rescued by neural stem cell transplantation [98].

Radiation-induced microvascular damage and neuroinflammation altering the microenvironment of the stem cell niche is another possible explanation for the mode of IR action in the hippocampus region. Dysregulated signaling in the hippocampal microenvironment may disturb complex differentiation processes and may suppress the physiological maturation of progenitor cells to their neuronal phenotype [92]. In the hippocampal microenvironment, the control of cell proliferation and survival in stem cell niches depends on balanced signaling networks and is thus an important prerequisite for the orderly development and maintenance of tissues [99,100]. The transcription factor cAMP response element-binding (CREB) plays a crucial role in the proliferation, differentiation and survival of neuronal stem/progenitor cells [101]. In response to genotoxic stress, CREB activation leads to the expression of various neuroprotective factors, thereby contributing to the survival of newborn neurons [102]. Disturbance of CREB functions in the brain can contribute to the development and progression of neurodegeneration.

### 4.3. Radiation-Induced Neurovascular Damage

Increasing research evidence indicates that the radiation effect is intensified by additional damage to the microvascular endothelium, leading to cerebrovascular inflammation with the potential disruption of the blood-brain barrier [103]. The blood-brain barrier plays a crucial role in maintaining tissue homeostasis by regulating trans-endothelial transport between brain parenchyma and bloodstream, and particularly by restricting the translocation of peripheral immune cells. This highly regulated but at the same time very fragile barrier system is organized by endothelial cells through interactions with pericytes and astrocytes and supports the trans-endothelial transport of vesicles within this neurovascular unit. In the acute setting, radiation-induced vascular damage is characterized by membrane destabilization of endothelial cells (detachment from basement membranes) and their induction of apoptosis leading to vascular leakage [104,105]. Radiation damage to the microvascular endothelium can promote cerebrovascular inflammation. Following IR exposure, endothelial cells acquire a pro-inflammatory phenotype with the expression of adhesion molecules, and the secretion of cytokines and chemokines, thereby facilitating the recruitment of immune cells to sites of tissue injury [106]. Disruption of the blood-brain-barrier results in the passage of systemic immune and inflammatory cells; their infiltration of the brain parenchyma enhances neuroinflammation [107]. Long-term damage to the vascular endothelium (as a result of inadequate repair of damaged endothelial cells) can result in tissue hypoxia and impaired metabolic homeostasis [107]. After radiation-induced brain damage, the structural and functional integrity of neurovascular networks may decline gradually within weeks through years post-IR and foster long-term cerebrovascular complications such as stroke [74]. Even repetitive low-dose IR (20× 0.1 Gy) of juvenile and adult mice induced long-lasting inflammatory responses, most pronounced in the hippocampal region of the juvenile brain, with an increased local blood flow and vascular permeability, as measured by MR imaging [97].

### 4.4. Radiation-Induced Neuroinflammation

Neuroinflammation is a multifaceted immune response involving numerous cell types (both within the CNS and in the peripheral circulation) with the aim of clearing the brain parenchyma from damaged cells or infectious agents. Microglia and astrocytes are considered key players in initiating the inflammatory response following injury to the CNS [108]. Dying or damaged cells within irradiated brain areas release cellular debris into the microenvironment, thereby priming local microglia and astrocytes to initiate an inflammatory cascade. Microglia cells reveal a large degree of heterogeneity in structure and shape, depending upon their activation state. While resting or surveilling, microglia cells have highly branched morphologies, activated microglia cells acquire de-ramified or amoeboid forms. Microglia cells remove dying cells and cellular debris through phagocytosis and together with astrocytes secrete inflammatory cytokines, chemokines, reactive oxygen and nitrogen species [109]. After a noxious stimulus, reactive astrocytes adopt a hypertrophic morphology, with swelling of cell bodies and elongated cell processes [97]. The pleiotropic responses of glial cells can result in both attenuation and enhancement of inflammatory responses dependent on the microenvironment, and ultimately these inflammatory reactions determine the extent of damage and subsequent regeneration.

Persistent activation of microglia and astrocytes is a key hallmark of chronic neuroinflammation [97]. Their prolonged activation leads to a vicious circle in which the secretion of pro-inflammatory factors leads to further neuronal damage, which in turn increases neurotoxicity and neurodegeneration. Overall, increasing evidence indicates that chronic neuroinflammation is a critical factor in radiation-induced brain damage.

### 4.5. Summary of Preclinical Studies in Rodents

Overall, research in pre-clinical rodent models provides basic insights into the pathophysiology of radiation-induced brain injury and the development of neurocognitive impairment. Basically, it turns out that the developing and immature brain is particularly vulnerable to the damaging effects of IR. The high content of progenitor cells, and that IR induces both the acute loss of neuroprogenitors through apoptosis and the perturbed microenvironment in stem cell niches, leading to disturbed proliferation and differentiation of neuroprogenitors, are fundamental mechanisms that explain the increased radiosensitivity of the immature brain. The extent of radiation damage is directly dependent on the developmental stage of neurogenesis and age-related increased cell loss of radiosensitive neuroprogenitors subsequently leads to pronounced neuroinflammatory and neurovascular responses. However, multiple factors are implicated in the etiology of radiation-induced cognitive impairment. Apart from the main causes presented above, there are other neurobiological processes such as impaired neuronal network connectivity, neurotransmitter imbalance, altered brain metabolism, etc., that may contribute to the pathogenesis of radiation-induced brain injury. For a detailed presentation of the molecular pathomechanisms of radiation-induced brain damage, reference is made to the following review article [27]. However, we are only at the beginning of the elucidation of these complex relationships in the pathophysiology of radiation-induced brain injury.

## 5. Conclusions

Radiotherapy-related neurocognitive impairment is a major clinical problem in neuro-oncology, especially in the treatment of brain tumors in children. Protective strategies aimed at minimizing damage to proliferative stem cell regions can significantly reduce neurocognitive damage and thus improve the quality of life, especially in children with a high probability of survival. In recent years, inverse planning and dose modulation with IMRT has enabled more precise targeting and sparing of critical structures in the brain. Further technological developments with more precise dose distribution will hopefully soon spare the critical hippocampus region in the brain so that neurocognitive impairment can be largely avoided [110]. Image guidance during radiation delivery and particle therapy with protons and carbon ions are also explored for additional improvements in precise dose distribution. Nevertheless, even RT procedures with stereotactic precision, produce scattered radiation to normal brain tissue outside the target areas [111], presenting an ongoing challenge in the radiation treatment of children. However, using modern conformal irradiation techniques with conventional fractionation and limited volumes, the expected risk of pronounced neurotoxicity for adult brain tumor survivors should be reasonably low. Nevertheless, optimizing radiation parameters is always a beneficial approach to reducing neurotoxicity and improving neurocognitive outcomes. Since high-dose fractional and total doses are more likely to induce cognitive impairment, conventional fractionation, and the lowest effective total dose according to evidence-based literature should be used in principle. Whenever possible, the IR volume of the brain should be limited by highly conformal techniques such as IMRT. If whole-brain RT is required, IMRT with hippocampal avoidance is expected to reduce the likelihood of severe adverse effects. Despite immense improvements in precision radiotherapy, there is an ongoing need for effective therapeutics in mitigating and treating radiation-induced brain injury. Molecular therapies to combat the progression and exacerbation of radiation-induced neuroinflammation must address the complexity of the inflammatory responses involved in complex brain tissue homeostasis. In general, a better understanding of the exact pathomechanisms will aid in the development of appropriate therapeutics to prevent neurocognitive sequelae in cerebral RT.

## Figures and Tables

**Table 1:** Atomic bomb survivors (n.s. = not specified).

Reference	Study Population (Location)	Sample Size	Type of Exposure	Age at Exposure	Brain Dose	Outcome	**Age at Outcome Measurement**
Wood, Johnson et al., 1967 [4]	atomic bomb survivors (Japan)	183	γ-rays and neutrons	in-utero	≤4 Gy	small head size, mental retardation	n.s.
Otake, Schull; 1984 [2]	atomic bomb survivors (Japan)	n.s.	γ-rays and neutrons	in-utero	≤4 Gy	forebrain damage, mental retardation	n.s.
Schull, Otake; 1986 [3]	atomic bomb survivors (Japan)	n.s.	γ-rays and neutrons	in-utero	≤4 Gy	mental retardation	n.s.
Otake, Schull; 1991 [9]	atomic bomb survivors (Japan)	1673	γ-rays and neutrons	in-utero	0.6–1.4 Gy	IQ decline, lower school performance	10–11 years
Yoshimaru, Otake et al., 1991 [8]	atomic bomb survivors (Japan)	929	γ-rays and neutrons	in-utero	≤4 Gy	lower school performance	n.s.
Ikenoue, Ikeda et al., 1993 [6]	atomic bomb survivors (Japan)	929	γ-rays and neutrons	in-utero	≤4 Gy	lower school performance	n.s.
Otake, Schull; 1993 [7]	atomic bomb survivors (Japan)	1473	γ-rays and neutrons	in-utero	≤4 Gy	small head size, mental retardation	9–19 years
Yoshimaru, Otake et al., 1995 [8]	atomic bomb survivors (Japan)	888	γ-rays and neutrons	in-utero	≤4 Gy	IQ decline, mental retardation	15–16 years
Yamada, Sasaki et al., 2002 [10]	atomic bomb survivors (Japan)	3113	γ-rays and neutrons	≥13 years	≤4 Gy	no neurocognitive dysfunction	adulthood
Yamada, Kasagi et al., 2009 [12]	atomic bomb survivors (Japan)	2286	γ-rays and neutrons	≥13 years	≤4 Gy	no increased risk of neurodegeneration	≥60 years
Yamada, Landes et al., 2016 [14]	atomic bomb survivors (Japan)	1844	γ-rays and neutrons	≥13 years	≤4 Gy	no increased risk of neurodegeneration	60–80 years
Yamada, Kato et al., 2021 [13]	atomic bomb survivors (Japan)	303	γ-rays and neutrons	in-utero	≤4 Gy	no increased risk of neurodegeneration	65–70 years
Ishihara, Kato et al., 2022 [11]	atomic bomb survivors (Japan)	469	γ-rays and neutrons	≤12 years	≤4 Gy	no increased risk of neurodegeneration	≥70 years

**Table 2:** Medically exposed children (m = months; y = years).

Reference	Study Population	Sample Size	Type of Exposure	Age at Exposure	Brain Dose	Outcome	Age at Outcome Measurement
Albert, Omran et al., 1966 [18]	tinea capitis (New York)	1908	X-ray RT	mean: 8 years	mean: 1.3 Gy	mental disorders, psychosis	21 years
Ron, Modan et al., 1982 [19]	tinea capitis (Israel)	10,842	X-ray RT	range: 1–15 years mean: 7 years	range: 0.7–1.6 Gy mean: 1.5Gy	IQ decline, lower school performance	24 years
Hall, Adami et al., 2004 [20]	haemangioma (Sweden)	2816	X-ray RT	range: 0–18 months mean: 7 months	range: 0–2.8 Gy mean: 0.02 Gy	neurocognitive dysfunction ≥0.25 Gy	18 years
Blomstrand, Holmberg et al., 2014 [21]	haemangioma (Sweden)	3030	RT (different IR qualities)	range: 0–18 months median: 5 months	range: 0–1.1 Gy median: 0.02Gy	hippocampus ≥0.2Gy → lower verbal skills	18 years
Nordenskjöld, Palme et al., 2015 [22]	maternal X-ray pelvimetry (Sweden)	1612	diagnostic X-ray	in-utero	estimated fetal dose: 0.0015 Gy	no effect on school performance	15 years
Salonen, Nyman et al., 2018 [23]	CT scan (Sweden)	147	diagnostic head CT	range: 6–16 years mean: 11 years	estimated dose 0.03–0.05 Gy	no cognitive dysfunction	18 years

**Table 3:** Childhood cancer survivors (n.s. = not specified).

Reference	Study Population	Sample Size	Type of Exposure	Age at Exposure	Dose	Outcome	Age at Outcome
Broadbent, Barnes et al., 1981 [39]	medulloblastoma (UK)	8	^60^Co RT (neuroaxis)	1–12 years	tumor: 43–50 Gy	mental retardation, younger children (≤2y) more affected	n.s.
Danoff, Cowchock et al., 1982 [40]	primary brain tumors (USA)	38	^60^Co RT	1–16 years	tumor: 40–65 Gy	mental retardation, younger children (≤3y) more affected	n.s.
Mulhern, Hancock et al., 1992 [42]	primary brain tumors (USA)	544	RT (local/ whole brain)	1–18 years	n.s.	IQ decline, younger children (≤4y) more affected	1–21 years after RT
Radcliffe, Bunin et al., 1994 [43]	medulloblastoma	24	cranial RT	1–20 years	n.s.	IQ decline, younger children (≤7y) more affected	2–4 years after RT
Skowrońska-Gardas, 1999 [44]	CNS tumors (Poland)	52	photon RT (neuroaxis)	1–3 years	tumor: 50 Gy neuroaxis: 30 Gy	mental retardation, younger children (≤3y) more affected	5 years after RT
Edelstein, Spiegler et al., 2011 [41]	medulloblastoma		photon RT		tumor: 50 Gy neuroaxis: 23 Gy	IQ decline, younger children (≤7y) more affected	≤40 years after RT
Yock, Yeap et al., 2016 [48]	medulloblastoma (USA)	59	proton RT (neuroaxis)	3–21 years	tumor: 54 Gy neuroaxis: 23 Gy	IQ decline, no age-dependent effect	7 years after RT
Ventura, Grieco et al., 2018 [47]	primary brain tumors (USA)	65	proton RT (local)	2–17 years	n.s.	IQ decline, no age-dependent effect	4–18 years after RT
Tso, Liu et al., 2019 [46]	germ cell tumors (Hong Kong)	25	cranial RT	7–18 years	tumor: 30–54 Gy	IQ decline, no age-dependent effect	1–12 years after RT
Stadskleiv, Stensvold et al., 2022 [45]	medulloblastoma (Norway)	50	photon RT (neuroaxis)	5–51 years	tumor: 44–56 Gy	IQ decline, no age-dependent effect	19 years after RT

**Table 4:** Prophylactic whole-brain radiotherapy.

Reference	Study Population	Sample Size	Type of Exposure	Age at Exposure	Brain Dose	Outcome	Age at Outcome Measurement
Meadows, Gordon et al., 1981 [36]	children with ALL (USA)	41	WBRT	2–15 years	24 Gy, fractionated	IQ decline; younger children more affected	1–3 years after RT
Twaddle, Britton et al., 1983 [55]	children with ALL (England)	23	WBRT	1–8 years	24 Gy, fractionated	IQ decline; younger children more affected	1–3 years after RT
Ladavas, Missiroli et al., 1985 [51]	children with ALL (Italy)	21	WBRT	2–9 years	24 Gy, fractionated	IQ decline; younger children (<5 years) more affected	1–3 years after RT
Said, Waters et al., 1989 [54]	children with ALL (Australia)	106	WBRT	1–8 years	18–24 Gy, fractionated	IQ decline; younger children more affected	1–13 years after RT
Chessells, Cox et al., 1990 [49]	children with ALL (England)	136	WBRT	1–12 years	18–24 Gy, fractionated	IQ decline, younger children (≤2 years) more affected	1–5 years after RT
MacLean, Noll et al., 1995 [52]	children with ALL (USA)	74	WBRT	3–7 years	18 Gy, fractionated	neuropsychological deficits	1 years after RT
Iuvone, Mariotti et al., 2002 [50]	children with ALL (Italy)	21	WBRT	1–12 years	18–24 Gy, fractionated	no age-dependent effect	4–12 years after RT
Reinhardt, Thiele et al., 2002 [53]	children with AML (Germany)	38	WBRT	0–18 years	12–18 Gy, fractionated	learning deficits, younger children more affected	4–11 years after RT
